# Case Report: longitudinal imaging in a patient with SMART syndrome after cranial radiotherapy

**DOI:** 10.3389/fonc.2026.1913288

**Published:** 2026-08-14

**Authors:** Da Bin Kim, Haroun Bel Hadj Jrad, Lena Bender, Jürgen Beck, Marcia Machein

**Affiliations:** 1 Department of Neurosurgery, University Medical Center Freiburg, Faculty of Medicine, University of Freiburg, Freiburg, Germany; 2 Department of Neuroradiology, University Medical Center Freiburg, Freiburg, Germany; 3 Department of Neurosurgery, Division of Presurgical Epilepsy Diagnostics, University Medical Center Freiburg, Freiburg, Germany

**Keywords:** case report, cranial radiotherapy, delayed radiation injury, gyriform enhancement, neuro-oncology survivorship, radiation-associated cavernoma, radiation-induced cortical vulnerability, smart syndrome

## Abstract

**Background:**

Stroke-like migraine attacks after radiation therapy (SMART) syndrome is a rare, delayed complication of cranial irradiation, characterized by migraine-like headache, seizures, and focal neurological deficits with transient unilateral gyriform cortical enhancement on MRI. It has usually been regarded as a single, isolated post-radiation event. Evidence on recurrence, prodromal manifestations, and any relationship with other radiation-induced vascular lesions remains limited. We hypothesize that SMART syndrome may represent a dynamic spectrum of radiation-induced cortical vulnerability rather than an isolated event.

**Case description:**

We present a 48-year-old woman treated for a right parietal anaplastic astrocytoma with surgical resection and cranial radiotherapy in 2001 at an outside institution abroad; the original radiotherapy parameters, including cumulative dose and fractionation, were not retrievable. Twenty-four years later she presented with focal seizure clustering, severe unilateral headache, and transient left-sided hemiparesis. MRI showed extensive unilateral gyriform cortical enhancement confined to the previously irradiated hemisphere. Lumbar puncture, performed after initial stabilization, after corticosteroid initiation, and several weeks after symptom onset, showed a non-inflammatory CSF profile that reduced the likelihood of major inflammatory mimics but did not by itself exclude infectious, autoimmune, or paraneoplastic disease. Short-interval follow-up MRI showed near-complete resolution of the enhancement. Retrospective review identified a similar but milder cortical enhancement episode nine years earlier, in 2016, which may represent an earlier SMART-like manifestation; a peri-ictal imaging change and a concurrent febrile illness at that time preclude a definite retrospective diagnosis. The patient also harbored multiple suspected radiation-associated cavernous malformations, predominantly within the previously irradiated right hemisphere.

**Conclusion:**

This longitudinal case supports the hypothesis that SMART syndrome may represent a spectrum of radiation-induced cortical vulnerability, with possible recurrence and earlier, milder manifestations. Its coexistence with these cavernous malformations is compatible with a shared substrate of chronic radiation-induced microvascular injury. We propose a multimodal diagnostic framework that combines clinical assessment, serial MRI, EEG, and exclusionary CSF analysis with short-interval follow-up imaging, to support conservative management and prevent unnecessary invasive interventions in neuro-oncology survivors, while recognizing that clinical deterioration should prompt re-evaluation regardless of initial findings.

## Introduction

Stroke-like migraine attacks after radiation therapy (SMART) syndrome is a late complication occurring years to decades after cranial radiotherapy, characterized by a triad of migraine-like headache, seizures, and focal neurological deficits with transient unilateral gyriform cortical enhancement on MRI (1–5). It has usually been regarded as a rare, isolated post-radiation event (1, 5), but growing evidence suggests it can recur and that earlier subtle cortical enhancement episodes may be prodromal manifestations of a broader continuum of radiation-induced cortical vulnerability (3, 5, 6). Because new enhancement in long-term survivors is often misread as tumor recurrence or encephalitis, it can prompt unnecessary invasive interventions such as biopsy or prolonged empiric antimicrobial therapy (3, 7, 8).

Baytok et al. (2026) recently proposed revised diagnostic criteria updating the original 2006 framework, reflecting clinical heterogeneity and recurrence (21). Its dynamic evolution has rarely been documented longitudinally, and its relationship to other delayed radiation-induced vascular lesions such as cavernous malformations remains unclear (3, 5, 6, 10–12). We present a case with nine years of imaging follow-up that includes a probable earlier, milder cortical enhancement episode, possibly an early manifestation within this spectrum although a peri-ictal etiology cannot be excluded, together with coexisting cavernous malformations (3, 6, 11, 12). The case generates the hypothesis of a spectrum model of radiation-induced cortical vulnerability and highlights the practical value of a multimodal work-up, including cerebrospinal fluid (CSF) analysis as an exclusionary component, to guide conservative management (3, 5, 7, 9, 13).

## Case presentation

### Patient information and oncological history

A 48-year-old woman underwent resection of a right parietal brain tumor in 2001 at an outside institution abroad, after presenting with language disturbance, headache, and facial hypoesthesia. The lesion was classified as an anaplastic astrocytoma (WHO grade III) and treated with postoperative cranial radiotherapy, described as two courses in the historical records. Original histopathology and detailed radiotherapy parameters (dose, fractionation, treated volume) were not retrievable; for context, WHO grade III glioma in that era was typically treated to around 60 Gy (14–16). She emigrated to Germany the following year and underwent cranial MRI surveillance about every three years without progression and had surgery for a right parietal wound-healing disorder in 2017. There was no family history of seizures, stroke, cavernous malformations, or hereditary neurovascular or neurocutaneous disorders, and no known genetic predisposition; her social and occupational history was unremarkable.

### Epilepsy course

The patient reported rare episodes of transient left-arm hypoesthesia with headache between 2010 and 2016, without an epileptological cause being considered at the time. Structural epilepsy was diagnosed only after a focal to bilateral tonic-clonic seizure in July 2016, witnessed to begin with left-arm clonic movements and left facial twitching, after which levetiracetam was started. She subsequently had stereotyped focal sensory seizures with hypoesthesia of the left hand and forearm, sometimes with weakness and occasionally triggered by systemic stressors. At an epilepsy visit in December 2025 she reported a recurrence in October 2025 and a cluster over several days in late November 2025; levetiracetam was then 2000 mg/day, with a recommendation to increase to 3000 mg/day (**Supplementary Table 1**). A full chronology from the 2001 radiotherapy to the most recent follow-up is given in **Table 1**.

**Table 1:** Timeline of the clinical course.

Timeframe	Event/intervention
~24 y before index (2001)	Resection of right parietal anaplastic astrocytoma (WHO III) plus two courses of cranial radiotherapy (parameters not retrievable), at an institution abroad
~15 & ~13 y before (≈2010 and 2013)	Transient left-arm sensory episodes lasting hours to a day, fully reversible; not recognized as epileptic at the time
~9 y before (2016)	First focal to bilateral tonic-clonic seizure; structural epilepsy diagnosed. MRI: band-like cortical enhancement (right parietal and peri-insular, leptomeningeal contact); LP unremarkable during a concurrent febrile infection; probable earlier SMART-like episode; levetiracetam started
~8 y before (2017)	Surgery for a right parietal wound-healing disorder
~7 y before (2018)	SMART syndrome first formally suspected
~6 y before (2019)	MRI: right temporal hemorrhage (~13 mm) plus multiple microcavernomas; suspected radiation-associated cavernous malformations
~4 y before (2021)	Routine surveillance MRI: stable, no progression
~3 y before (2022)	Further SMART-suspected episode: acute left-arm focal seizure with holocephalic headache; MRI (regional hospital) no fresh bleed or tumor recurrence; levetiracetam increased; subsequently seizure-free
~10 & ~8 months before	Surveillance MRIs: known cortical FLAIR changes stable, no new pathology
Months before index	Recurrent focal self-limiting seizures; levetiracetam up-titrated
Day 0 (index presentation)	Several days of focal seizure cluster, right-sided headache (7–8/10), and worsening left brachiofacial paresis
Day ~0–2	Admission to a regional hospital: infection screen negative; EEG (right temporal slowing, no epileptiform activity); dexamethasone 12 mg/day started
Day ~1	MRI: extensive unilateral gyriform cortical enhancement in the irradiated right hemisphere plus a small DWI infarct in the right insula/external capsule
Day ~5–9	Neurosurgery brain-tumor clinic and tumor board (suspected recurrent SMART); epileptology (levetiracetam up-titrated toward 3000 mg/day)
~Week 6	Neurology admission and LP: 1 cell/µL, protein 194 mg/L, IgG index 0.51, OCB negative; antibody panels unremarkable; left paresis resolved on exam
~Week 8	Follow-up MRI: near-complete resolution (punctate residua), no diffusion restriction, no hyperperfusion, no tumor progression; next surveillance MRI in 1 year
Weeks–months after index	Four self-limited headache episodes (December–January) without seizure-suggestive semiology, each promptly responsive to oral corticosteroids
~10 weeks & ~7 months after index	Epileptology follow-ups: sustained seizure freedom since the index episode (after levetiracetam 1 to 3 g/day)

Timing is given relative to the index (most recent) presentation, with calendar years shown for distant milestones.

### Probable earlier cortical enhancement episode (August 2016)

An MRI in early August 2016, shortly after the first seizure, showed band-like gyriform cortical enhancement in the right parietal and peri-insular cortex with leptomeningeal contact and subtle diffusion abnormalities; dynamic susceptibility contrast (DSC) perfusion showed no focal hyperperfusion or pathological elevation of relative cerebral blood volume (rCBV). The differential then included post-ictal change, subacute ischemia, and tumor recurrence, and the enhancement resolved spontaneously. Clinically, the episode comprised the triad of a persistent temporal headache, a first focal to bilateral tonic-clonic seizure, and a prolonged left-arm sensory deficit, and a lumbar puncture at the referring hospital was unremarkable. Two features nonetheless keep it short of a definite retrospective diagnosis: it occurred in immediate peri-ictal proximity and coincided with a febrile respiratory infection, so neither a peri-ictal imaging change nor an infectious contribution can be excluded. It is therefore best classified as a probable earlier SMART-like episode rather than definite SMART (**Supplementary Figure 1**) (3, 6).

### Delayed vascular sequelae (August 2019)

In August 2019, MRI showed a subacute intraparenchymal hemorrhage of about 13 mm in the right temporal lobe (**Supplementary Figure 2C**) together with multiple punctate susceptibility foci consistent with small cavernous malformations (**Supplementary Figures 2A, B**). Given the long latency after irradiation and the predominantly ipsilateral distribution, these were considered most consistent with radiation-associated rather than sporadic lesions, although the foci were bihemispheric (subcortical and central white matter, sparing the basal ganglia) with a clear right-sided predominance, which precludes definitive attribution to radiotherapy in the absence of the original treatment field (11, 12). Such lesions may mark chronic microvascular remodeling, aligning with the vulnerability implicated in SMART syndrome (10, 12). Cerebral amyloid angiopathy was considered but judged less likely given the right-sided predominance and the patient’s age. Because some foci lie outside the presumed irradiated field, we describe them as suspected radiation-associated cavernous malformations rather than asserting a definitive cause.

### Acute episode consistent with SMART syndrome (late November 2025)

**Figure 1:**
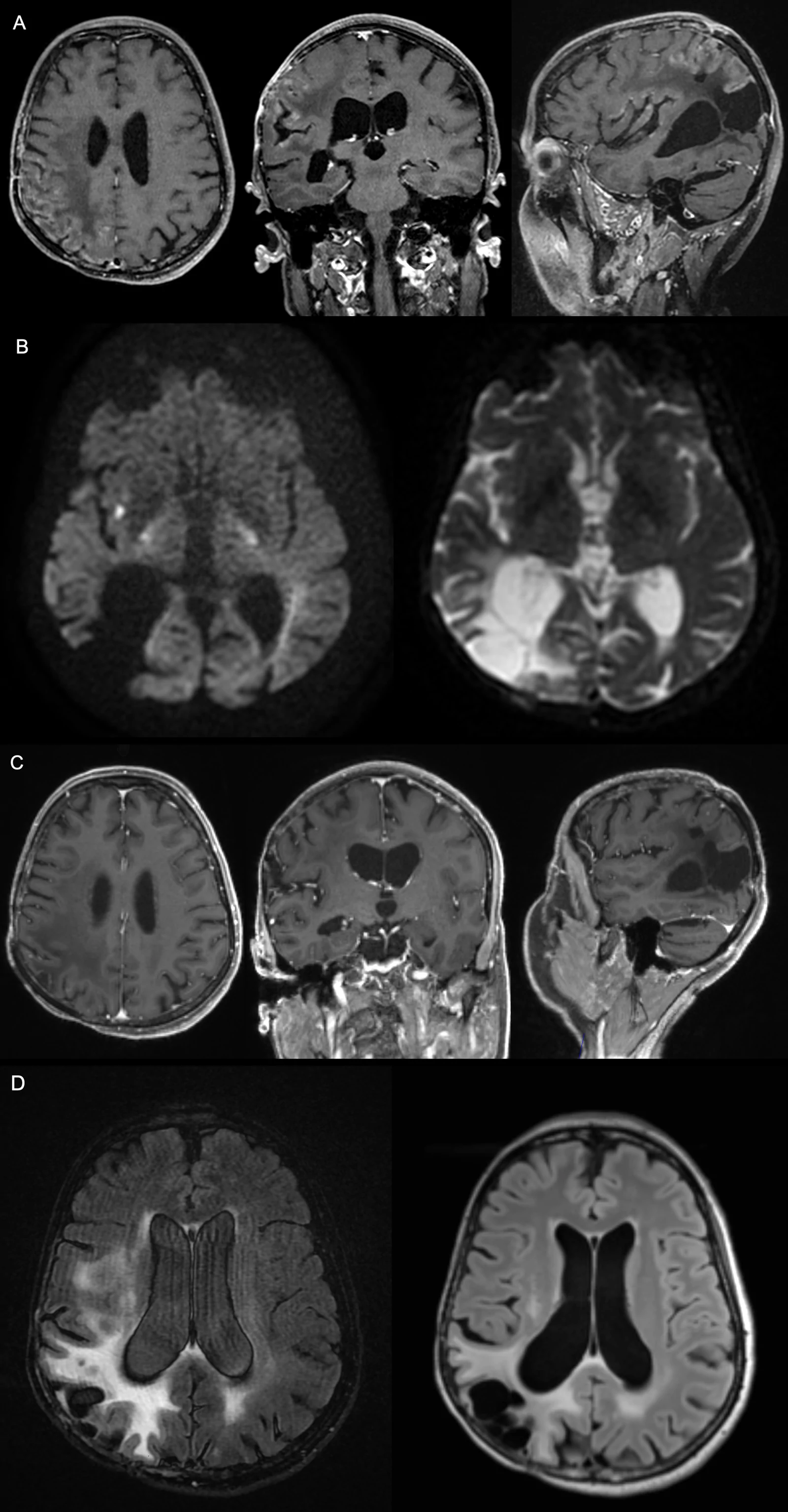
Acute SMART episode and its reversibility. **(A)** Acute T1 post-contrast MRI (November 2025), axial, coronal, and sagittal planes, showing extensive unilateral gyriform cortical enhancement within the previously irradiated right hemisphere. **(B)** Acute diffusion-weighted imaging showing a small focus of restricted diffusion in the right insular and external-capsule region, an incidental finding radiologically distinct from the cortical enhancement. **(C)** Follow-up T1 post-contrast MRI (mid-January 2026), same planes, showing near-complete regression with only minimal punctate residual enhancement. **(D)** T2-FLAIR comparison, acute (left) versus follow-up (right), showing resolution of the cortical swelling and hyperintensity.

In late November 2025 she developed several days of frequent focal seizures, right-sided headache, and progressive left facial weakness with mild left sensorimotor hemiparesis. The pain was right-sided, throbbing, severe (7–8 on a 0–10 numeric rating scale), and aggravated by routine activity, resembling migraine. Within ICHD-3, which does not recognize SMART as a distinct entity, it is best regarded as a secondary headache attributed to SMART syndrome rather than primary migraine, since it arose acutely from a known intracranial process, lasted several days, and did not meet the full criteria for migraine without aura; we therefore describe it as migraine-like, consistent with the phenotype usually reported in SMART syndrome. At neurosurgical evaluation in December 2025 she was already receiving dexamethasone 12 mg/day, with improving headache but persistent mild left brachiofacial-predominant weakness, which is non-specific and can also occur in inflammatory or neoplastic mimics. MRI showed extensive unilateral gyriform cortical enhancement within the irradiated right hemisphere and a small acute ischemic lesion in the right external capsule (**Figures 1A, B**) (1–3).

### Investigations

Electroencephalography showed persistent focal slowing over the right centro-parietal-temporal region without epileptiform discharges, compatible with structural change; a normal or non-epileptiform interictal EEG does not, however, exclude a seizure-related imaging abnormality. A breach effect was also noted at the prior craniotomy site, with prominent high-amplitude beta activity and sharply contoured waveforms, attributable to the craniotomy defect rather than to cortical dysfunction. DWI showed restricted diffusion in the right insula and external capsule, an incidental finding on the acute MRI (**Figure 1B**). Although it lies near the descending motor pathways and may have contributed to the transient left-sided weakness, it is radiologically distinct from the reversible gyriform cortical enhancement, so the two are best regarded as concurrent rather than causally identical. It was read in more than one way during the work-up, first as possibly paraneoplastic and later as a small acute ischemic lesion. Because no dedicated vascular imaging (intracranial or cervical vessel imaging or a cardioembolic evaluation) was performed during the acute admission, an unrelated small-vessel ischemic event, radiation-associated vasculopathy, and an event tied to the acute episode cannot be reliably distinguished; we note this as a limitation. As the left-sided weakness resolved completely while the infarct persisted, the reversible cortical process rather than the infarct was most likely the principal driver of the transient deficit, although a contribution from the insular and external-capsule lesion cannot be fully excluded. Because these features overlap with infectious (including herpes) and autoimmune encephalitis and neoplastic leptomeningeal disease, a lumbar puncture was performed to help exclude inflammatory etiologies (3, 5, 7, 13).

During the acute episode, aminotransferases were markedly elevated, most likely reflecting a transient hepatocellular process in the setting of acute systemic illness; creatine kinase remained normal, which argues against a primary skeletal-muscle (rhabdomyolytic) source. The values normalized without specific hepatological treatment, confirming the transient nature of the injury (**Supplementary Table 2**).

CSF was sampled during a dedicated diagnostic admission, approximately six weeks after symptom onset and after corticosteroid initiation (dexamethasone 12 mg/day, started at presentation), to help exclude high-consequence inflammatory mimics. It showed 1 cell/µL (90% lymphocytes), protein 194 mg/L, an albumin quotient of 3.0 × 10^-3^, an IgG index of 0.51, and negative oligoclonal bands (a single identical CSF/serum band, non-specific), a non-inflammatory profile without barrier dysfunction or intrathecal synthesis. Paraneoplastic anti-neuronal and autoimmune-encephalitis antibody panels available at discharge were unremarkable (**Supplementary Table 3**). This profile supported de-escalation of infectious and inflammatory differentials and conservative management without invasive procedures. Because CSF was obtained after corticosteroids and several weeks after onset, however, it supported rather than guided management, and a non-inflammatory profile does not by itself exclude infectious, autoimmune, or paraneoplastic disease (7, 13).

### Outcome and follow-up

**Figure 2:**
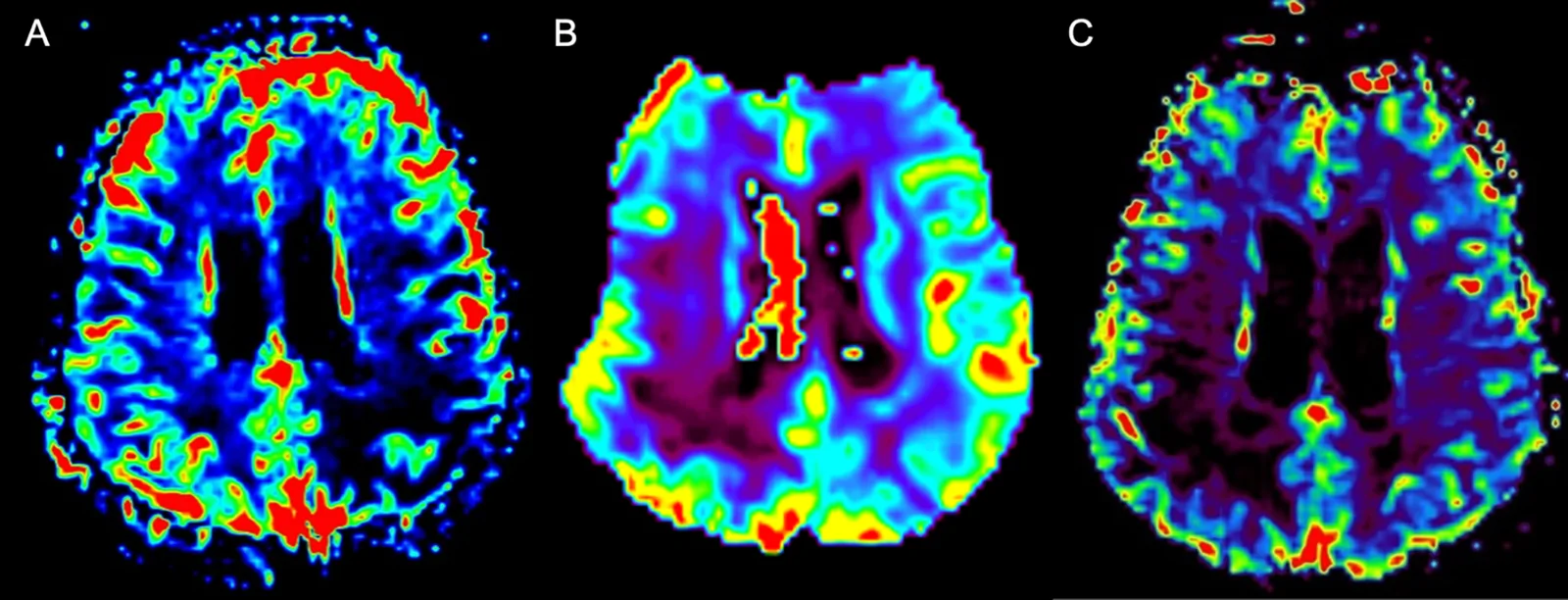
Dynamic susceptibility contrast (DSC) perfusion, shown as relative cerebral blood volume (rCBV) maps, at three time points: **(A)** the precursor episode (August 2016), **(B)** the acute SMART episode (November 2025), and **(C)** follow-up (January 2026). At each time point rCBV is normal to mildly reduced without focal hyperperfusion, arguing against a tumor-associated hyperperfusion pattern.

Follow-up MRI in mid-January 2026 showed near-complete resolution of enhancement in the right parietal lobe, with only punctate residual enhancement, no diffusion restriction, no pathological hyperperfusion, and no imaging evidence of tumor progression (**Figures 1C, D**, **2**). The patient had complete resolution of headache and focal deficits on corticosteroid therapy and levetiracetam optimization (3000 mg/day). Epileptological review at approximately 10 weeks and 7 months confirmed sustained seizure freedom after augmentation of levetiracetam from 1 to 3 g/day. Four self-limited headache episodes without seizure-suggestive semiology occurred over the following two months, each responding promptly to oral corticosteroids, and no seizures occurred after the index episode. The overall course supported a diagnosis of SMART syndrome (1–3, 9).

## Discussion

### SMART syndrome as a proposed spectrum of cortical vulnerability

The case leads us to hypothesize that SMART syndrome may be not a single, isolated post-radiation event but a dynamic spectrum of cortical vulnerability, with possible prodromal manifestations and recurrence (3, 5, 6). The 2016 band-like enhancement resolved spontaneously and was first read within a broad differential. After the 2025 episode, with its extensive gyriform enhancement and typical reversibility, the earlier episode can be cautiously re-interpreted as possibly fitting this spectrum, although a peri-ictal imaging change remains a plausible alternative (20); our reading does not depend on classifying the 2016 event as definite SMART (3, 6). In this patient the clinical pattern was recurrent over more than a decade: transient left-arm sensory disturbance with headache (around 2010 and 2013), the 2016 episode, a further SMART-suspected episode in 2022, and the 2025 presentation, all confined to the irradiated right hemisphere, supporting without proving the proposed spectrum model. The recurring peri-ictal and sometimes infective context is exactly why each episode is best read cautiously, and the criteria recently proposed by Baytok et al., which explicitly allow recurrent episodes and incomplete recovery, reinforce this reading (21).

Beyond imaging, the clinical semiology itself provides convergent evidence for cortical dysfunction localized to the irradiated field. The stereotyped focal sensory seizures, with left-hand hypoesthesia and occasional weakness, are consistent with involvement of the right intraparietal sulcus and inferior parietal cortex along the supramarginal gyrus, regions responsible for higher-order somatosensory processing and integration of tactile information from the contralateral limb. Dense cortico-cortical connectivity between these parietal areas and the adjacent motor cortex likely allowed the rapid propagation seen during the initial focal to bilateral tonic-clonic seizure in 2016. Even the earliest sensory episodes, which predated both the first recognized seizure and the first documented cortical enhancement, are consistent with intermittent dysfunction of this same parietal cortex.

### Multimodal diagnostic strategy: CSF as one exclusionary component

A central challenge is diagnostic triage: this triad with marked cortical enhancement overlaps with encephalitis and other inflammatory disorders that need urgent treatment. The migrainous quality of the headache is itself informative, since a non-migrainous (for example, tension-type) headache would lower the pre-test probability of SMART syndrome. Here the diagnosis rested on convergence across modalities (clinical course, serial MRI, EEG, DSC perfusion, and CSF) rather than any single test. Lumbar puncture served to reduce the probability of inflammatory alternatives rather than to prove SMART syndrome (5, 7, 13); the non-inflammatory CSF and unremarkable antibody diagnostics, with the characteristic enhancement and its rapid regression on short-interval follow-up MRI, supported conservative management and reduced pressure toward biopsy (2, 3, 7). Because a non-inflammatory profile does not categorically exclude early viral, autoimmune, or paraneoplastic disease, clinical deterioration or atypical imaging should prompt escalation regardless of CSF findings. Beyond the two-case series of Baytok et al. (21), the central contribution of this report is nine years of longitudinal, serial-MRI documentation of the dynamic evolution and reversibility of the cortical process, within which CSF plays a supporting, exclusionary role; the key distinguishing features are summarized in **Supplementary Table 4**.

### Differentiation from tumor recurrence

The acute enhancement was exclusively gyriform and confined to the cortical ribbon within the irradiated hemisphere, without nodular, mass-like, or expansile components or mass effect, features that argue against tumor recurrence. It was first interpreted at the referring center as tumor recurrence, illustrating the pitfall this report addresses; the near-complete reversibility on follow-up MRI clarified the diagnosis. Because no histopathological confirmation was obtained, recurrence cannot be excluded histologically, but demonstrated reversibility is not a feature of neoplastic progression and argues against it.

DSC perfusion at three time points (August 2016, November 2025, and January 2026) consistently showed normal to mildly reduced rCBV without focal hyperperfusion (**Figure 2**). The small external-capsule infarct is best regarded as a concurrent but radiologically distinct vascular event rather than a core manifestation of SMART syndrome. Together with the normal perfusion, the rapid regression supports a reversible radiation-related cortical process (**Supplementary Table 4**), reinforcing serial MRI as a key decision point that can prevent overtreatment and unnecessary procedures (2, 3, 5, 9).

### Differential diagnosis

A broad differential merits consideration in an irradiated survivor presenting with cortical signal change and gyriform enhancement. Infection (including herpes), autoimmune or paraneoplastic encephalitis, tumor recurrence, and leptomeningeal carcinomatosis were addressed above through CSF, serial MRI, and reversibility, and ischemic or vascular processes remained relevant given the concurrent external-capsule lesion. Posterior reversible encephalopathy syndrome (PRES) was unlikely without its characteristic posterior, vasogenic distribution or a hypertensive context, and mitochondrial encephalomyopathy, lactic acidosis, and stroke-like episodes (MELAS) was improbable given the late-onset, radiation-associated setting and the absence of a maternal-inheritance or systemic phenotype. Peri-ictal pseudoprogression (PIPG) is particularly relevant: in previously irradiated patients, seizures can produce transient, reversible cortical changes, including gyriform enhancement and diffusion abnormalities, that closely mimic tumor progression or SMART syndrome (20, 22, 23); the absence of focal hyperperfusion also argues against an active ictal process. Because this patient’s episodes clustered around seizures, PIPG is an important and not fully excludable alternative, most notably for the 2016 event, and we regard it as part of the same radiation-related continuum that future work should aim to separate from SMART.

### Suspected radiation-associated cavernomas and a hypothesized pathophysiological model

Cavernous malformations arising years after cranial radiotherapy are a recognized delayed complication (11, 12). Here the long latency and predominantly irradiated-hemisphere distribution point toward a radiation-associated rather than sporadic origin, though the missing treatment field precludes proof. A direct causal link with SMART syndrome cannot be drawn from a single case; their co-occurrence within the same irradiated hemisphere prompts our hypothesis of a shared substrate of chronic radiation-induced microvascular vulnerability, in which the cavernomas are a structural expression of microvascular remodeling and fragility and SMART a functional, reversible expression of endothelial and blood-brain barrier dysfunction affecting the cortex (3, 10, 12).

### Therapeutic considerations

For empirical acute therapy, short courses of high-dose corticosteroids are frequently used and may relieve symptoms (6, 9, 17–19); reported regimens include intravenous methylprednisolone and steroid pulses (17, 18). In our patient, clinical and radiological improvement under ongoing dexamethasone was consistent with a possible acute benefit; a recent report similarly documented steroid response in SMART occurring 36 years after irradiation (23). Steroids have also been linked to incomplete recovery in some series, so risks and benefits should be weighed individually (9, 19). No disease-specific therapy is established; calcium-channel blockers such as verapamil have been used, presumably targeting cerebrovascular autoregulation and cortical excitability, but responses are inconsistent and they are best regarded as adjunctive (6, 19).

**Figure 3:**
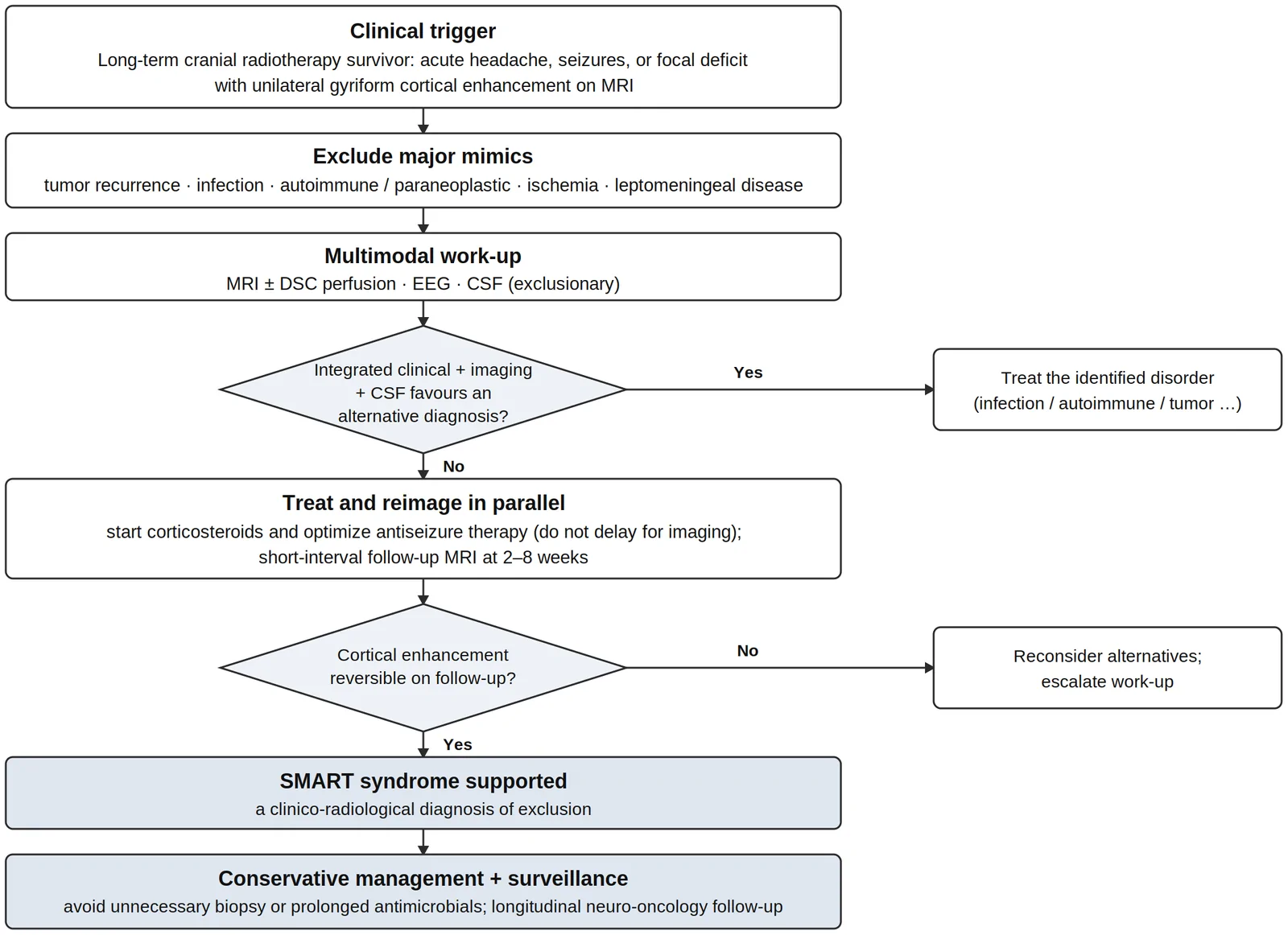
Multimodal diagnostic and management algorithm for suspected SMART syndrome in long-term cranial radiotherapy survivors. Boxes denote assessments or actions and diamonds denote decision points, running from clinical suspicion through exclusion of mimics and multimodal work-up to short-interval follow-up MRI and conservative management. Symptomatic treatment is started in parallel and is not delayed pending imaging. CSF, cerebrospinal fluid; MRI, magnetic resonance imaging.

Symptomatic treatment (corticosteroids and optimization of antiseizure therapy) is started promptly on clinical suspicion and is not withheld pending imaging; in this patient both began at presentation. Short-interval follow-up MRI serves to confirm reversibility and exclude progression, guiding de-escalation of invasive work-up rather than gating the start of treatment. We therefore propose a pragmatic, multimodal diagnostic and management algorithm for suspected SMART syndrome in long-term radiotherapy survivors presenting with acute neurological symptoms and unilateral gyriform enhancement (**Figure 3**), prioritizing exclusion of major mimics and confirmation of reversibility to support conservative management and avoid unnecessary biopsy. It should be understood as expert opinion derived from a single case rather than a validated algorithm, intended to structure clinical reasoning and requiring prospective evaluation before wider use.

### Patient perspective

The patient reported that the sudden severe headache and worsening weakness were frightening, given her long history of epilepsy after brain tumor treatment. She was relieved the symptoms resolved and valued a clear explanation, noting that understanding the cause reduced her anxiety about tumor recurrence, and she remains committed to regular follow-up and to taking her medication.

## Limitations

Several limitations apply. First, original radiotherapy documentation (dose, fractionation, treated volume) was not retrievable, which makes attribution of the cavernous malformations to radiotherapy presumptive and meant that the ≥50 Gy threshold in the recently proposed criteria could not be independently confirmed (21). Second, the reading of the 2016 episode as a probable earlier manifestation is retrospective, with a peri-ictal imaging change an equally plausible explanation (20). Third, as a single-case observation, the findings cannot establish causality or generalizability, and the proposed spectrum model needs validation in larger cohorts. Fourth, perfusion was interpreted qualitatively from rCBV maps, as quantitative ratios were unavailable. Fifth, the acute external-capsule infarct and its possible contribution to the deficits could not be fully characterized with the available vascular investigations. Finally, the multimodal framework is a practical tool rather than a validated criterion and should be tested prospectively.

## Conclusion

This longitudinal case generates the hypothesis that SMART syndrome may represent a spectrum of radiation-induced cortical vulnerability, with possible recurrence and earlier, milder manifestations, while acknowledging that the earlier episode may instead reflect a peri-ictal change. The co-occurrence of suspected radiation-associated cavernomas is compatible with a shared background of delayed microvascular injury. Our multimodal framework offers a practical way to prevent unnecessary invasive interventions while maintaining diagnostic safety and recognizing SMART syndrome as a possible spectrum disorder is increasingly important as the population of long-term neuro-oncology survivors grows.

## Data Availability

The original contributions presented in the study are included in the article/**Supplementary Material**. Further inquiries can be directed to the corresponding author.
