# Supplementary material for: Case Report: longitudinal imaging in a patient with SMART syndrome after cranial radiotherapy
Source: Front Oncol. 2026 Aug 14;16:1913288. doi: 10.3389/fonc.2026.1913288 (PMC13521836; doi:10.3389/fonc.2026.1913288)
Supplement: Supplementary file 1 [file Table1.docx]

**Supplementary Material**

Longitudinal Imaging in a Patient With SMART Syndrome After Cranial Radiotherapy

**Supplementary Figure Legends**

**Supplementary Figure 1. Probable earlier cortical enhancement episode (2016).** (A) T1 post-contrast MRI (2016) showing band-like gyriform cortical contrast enhancement in the right parietal and peri-insular cortex with leptomeningeal contact, shortly after the first seizure. (B) Follow-up MRI demonstrating spontaneous resolution of the enhancement. The episode occurred in immediate peri-ictal proximity and during a concurrent febrile illness, and is therefore regarded as a probable earlier SMART-like manifestation rather than definite SMART.

**Supplementary Figure 2. Suspected radiation-associated cavernomas and hemorrhage.** (A) SWI/T2* (2016) showing punctate susceptibility foci compatible with microcavernomas, distributed bihemispherically (subcortical and central white matter, sparing the basal ganglia). (B) SWI/T2* (August 2019) showing an interval increase and redistribution of the susceptibility foci. (C) T1 and SWI/T2* (August 2019) demonstrating a right temporal intraparenchymal hemorrhage (~13 mm), consistent with cavernoma-related bleeding. Attribution to radiotherapy remains presumptive.

**Supplementary Tables**

**Supplementary Table 1. Antiseizure medication timeline (levetiracetam)**

| **Time point** | **Total daily dose** | **Rationale / notes** |
| --- | --- | --- |
| July 2016 (after first seizure) | Levetiracetam started | Initiated after the first focal-onset seizure with secondary generalization (2016) |
| End of September 2025 | 750–0–750 mg/day (1500 mg/day) | Up-titration after recurrent focal seizures |
| Early December 2025 | 1000–0–1000 mg/day (2000 mg/day) | At epilepsy clinic; recommendation to titrate to 3000 mg/day |
| January 2026 (discharge) | 1500–0–1500 mg/day (3000 mg/day) | Documented at discharge |

**Supplementary Table 2. Blood laboratory values across the clinical course, with local reference ranges**

Values are shown at two representative time points: the acute phase (approximately two weeks after the index presentation) and convalescence (approximately six weeks after the index presentation). Reference ranges are those of the reporting central laboratory; clinical-chemistry analytes were measured in lithium-heparin plasma and hematology analytes in whole blood, so electrolyte ranges (for example potassium, 3.4–4.9 mmol/L) reflect plasma rather than serum values. H = above / L = below the reference range.

| **Analyte** | **Unit** | **Acute phase (~2 weeks)** | **Convalescence (~6 weeks)** | **Reference range** |
| --- | --- | --- | --- | --- |
| Leukocytes | 10³/µL | 10.23 | 6.36 | 4.0 – 10.4 |
| Platelets | 10³/µL | 496 (H) | 447 (H) | 176 – 391 |
| Haemoglobin | g/dL | 13.6 | 13.9 | 11.6 – 15.5 |
| Sodium | mmol/L | 135 (L) | 145 | 136 – 145 |
| Potassium | mmol/L | 4.0 | 3.7 | 3.4 – 4.9 |
| Urea | mg/dL | 16 (L) | 14 (L) | 16.6 – 48.5 |
| Creatinine | mg/dL | 0.51 | 0.55 | 0.51 – 0.95 |
| eGFR (CKD-EPI) | mL/min/1.73m² | 114 | 111 | — |
| AST (GOT) | U/L | 244 (H) | 27 | 10 – 35 |
| ALT (GPT) | U/L | 648 (H) | 36 (H) | 10 – 35 |
| Gamma-GT | U/L | 135 (H) | 52 (H) | < 40 |
| Creatine kinase (CK) | U/L | 39 | 53 | < 170 |
| C-reactive protein | mg/L | 10.3 (H) | 0.6 | < 5 |
| TSH | µIU/mL | 0.67 | 0.87 | 0.27 – 4.20 |

The marked but transient elevation of aminotransferases (AST/ALT) and gamma-GT during the acute phase normalized or near-normalized by convalescence without specific hepatologic intervention. Creatine kinase remained within the reference range at both time points, which argues against a primary skeletal-muscle (rhabdomyolytic) source and favors a transient hepatocellular process in the context of the acute systemic illness. Renal function remained normal throughout.

**Supplementary Table 3. CSF results, reference values, and diagnostic implications**

| **CSF parameter** | **Result** | **Reference (normal)** | **Interpretation** |
| --- | --- | --- | --- |
| Cell count | 1 cell/µL (90% lymphocytes) | < 5 cells/µL | No pleocytosis; lowers likelihood of active infectious/autoimmune encephalitis |
| Total protein | 194 mg/L | 150 – 450 mg/L | Non-inflammatory range in the clinical context |
| Albumin quotient (Q-Alb) | 3.0 × 10⁻³ | < 8 × 10⁻³ (age-dependent) | No blood–CSF barrier dysfunction |
| IgG index | 0.51 | < 0.7 | No intrathecal IgG synthesis |
| Oligoclonal bands (CSF/serum) | Negative (1 identical band, non-specific) | Negative | No intrathecal IgG synthesis |
| Autoimmune-encephalitis and paraneoplastic (antineuronal) antibody panels | Negative (both panels; results available at discharge) | Negative | Do not support autoimmune or paraneoplastic encephalitis in this clinical context |

CSF immunoglobulin indices are interpreted against Reiber reference diagrams rather than fixed cut-offs; the reference column gives conventional normal limits for general guidance. All values in this patient were within normal limits. Lumbar puncture was performed approximately six weeks after symptom onset (after corticosteroid initiation and well beyond the acute phase), so CSF supported, rather than guided, management.

**Supplementary Table 4. Differential diagnosis of unilateral gyriform cortical enhancement in long-term irradiated patients**

| **Feature** | **SMART syndrome** | **Tumor recurrence** | **Peri-ictal change / PIPG** | **Infectious / autoimmune encephalitis** |
| --- | --- | --- | --- | --- |
| Enhancement | Thin gyriform, strictly cortical | Nodular / infiltrative ± white matter | Gyriform cortical | Cortical ± leptomeningeal |
| Mass effect | None | Often present | Minimal | Variable |
| DWI | Usually negative | Variable | Transient cortical restriction | Often positive |
| Perfusion (rCBV) | Normal or reduced | Elevated | Normal / mild ↑ | Variable |
| FLAIR | Cortical hyperintensity | Mass-like, white-matter infiltration | Cortical ± subcortical | Often multifocal |
| Evolution | Regression in weeks | Progressive | Resolves in days | Variable |
| CSF | Typically non-inflammatory | Normal or ↑ protein; cytology may be positive | Normal | Often inflammatory |
| Clinical course | Reversible over weeks | Progressive | Transient | May worsen without treatment |

Infectious and autoimmune encephalitis are grouped for brevity; CSF profiles may differ substantially between subtypes. Posterior reversible encephalopathy syndrome (PRES) and MELAS are considered in the main-text Discussion (Differential Diagnosis) and are distinguished on clinical and distributional grounds. PIPG, peri-ictal pseudoprogression; rCBV, relative cerebral blood volume.
